# What Self-Management Skills Do Turkish Caregivers Have in Caring for People with Dementia? Results of a Qualitative Survey

**DOI:** 10.3390/healthcare12121187

**Published:** 2024-06-12

**Authors:** Yüce Yilmaz-Aslan, Kübra Annac, Tugba Aksakal, Hüriyet Yilmaz, Sibille Merz, Diana Wahidie, Oliver Razum, Patrick Brzoska, Hürrem Tezcan-Güntekin

**Affiliations:** 1 Department of Epidemiology & International Public Health, School of Public Health, Bielefeld University, 33615 Bielefeld, Germany; yuece.yilmaz-aslan@uni-wh.de (Y.Y.-A.); kuebra.annac@uni-wh.de (K.A.); tugba.aksakal@uni-wh.de (T.A.); tezcan@ash-berlin.eu (H.T.-G.); 2 Health Services Research, Faculty of Health, School of Medicine, Witten/Herdecke University, 58455 Witten, Germany; diana.wahidie@uni-wh.de (D.W.); patrick.brzoska@uni-wh.de (P.B.); 3 Intercultural Specialized Services of the Arbeiterwohlfahrt UB Gelsenkirchen/Bottrop, 45881 Gelsenkirchen, Germany; hueriyet.yilmaz@awo-gelsenkirchen.de; 4 Department of Health and Education, Alice Salomon University of Applied Sciences, 12627 Berlin, Germany; merz@ash-berlin.eu

**Keywords:** self-management, home care, family caregivers, dementia, diversity

## Abstract

Family caregivers can be overwhelmed by the care they provide within the family without external support. The development of self-management skills and the associated ability to actively and responsibly manage one’s own health or illness situation therefore plays a vital role in the home care of people living with dementia. As part of an individualized intervention for family caregivers of people of Turkish origin with dementia, existing self-management skills were examined through qualitative interviews to gain insight into health literacy and empowerment in caregiving and in interviewees’ own practices to maintain their health. Ten caregivers of Turkish origin who were responsible for family members living with dementia were interviewed using problem-centered interviews. We found that the target group has very heterogeneous self-management competencies, which are based, on the one hand, on existing supportive resources and, on the other hand, on diverse care-specific, psychosocial and life-world challenges in intrafamily care that have not been overcome. Self-management skills in family caregivers are influenced by a complex interplay of both available resources that support these skills and challenging caregiving situations. This dynamic combination of resources and challenges results in varying levels of self-management ability among family caregivers. Strengthening resources can help caregivers to meet the challenges resulting from caregiving and to expand their self-management competencies. There is great need for action in promoting self-management skills among Turkish caregivers of people living with dementia in home care. Interventions to promote self-management skills must take into account the individual resources of those affected as well as their social and cultural diversity.

## 1. Introduction

In 2022, 23.8 million people of the population in Germany (28.8%) had a history of migration (“people with a history of migration” refers to immigrant and non-immigrant foreigners, immigrant and non-immigrant naturalized citizens, (late) repatriates and the descendants of these groups born as Germans) [1]. People of Turkish origin represent the largest group within this population, comprising a total of about 3 million [2]. In the future, not only will the proportion of people with a history of migration increase significantly [3], but so will the proportion of immigrant older people. Migrants who came to Germany as part of the recruitment agreements (in 1961, the Federal Republic of Germany and Turkey signed a recruitment agreement that established the framework for the organized recruitment and employment of Turkish workers in Germany, significantly impacting cross-border labor mobility during that era) in the 1960s are now reaching an age at which age-related chronic diseases and the need for long-term care become more frequent [4].

Dementia is one of the most common and severe neurodegenerative diseases in old age [5]. As a result of the progressive impairment of brain functions and the associated loss of autonomy, dementia and dementia-related diseases represent one of the main causes for needing long-term care [6]. Given the changing age structure of the population due to demographic changes and the increasing number of chronically ill people, an increase in the need for long-term care is also to be expected in the population with a migration background in the coming years [7].

People with a history of migration who need care are almost exclusively cared for by relatives in their own homes [8]. This population makes little or no use of institutional care services. Without external support, family caregivers may be overwhelmed with care within the family in a variety of ways, which may lead to their own illness in the long term [9,10]. There is therefore a great need to support family caregivers by promoting their self-management skills.

The ability to recognize, define and evaluate problems and to make sustainable decisions as part of self-management competencies can assist family caregivers. Effective use of resources, building and maintaining sustainable relationships with professional stakeholders, and developing self-management skills are also beneficial [11]. These self-management competencies were originally defined in the context of chronically ill patients [11,12]. Family caregivers may have to adapt to changes and new challenges in daily life similar to the onset of their own potential chronic illness and may experience a state of “passive suffering” when close relatives become dependent on care [13]. Therefore, self-management skills are also important for family caregivers. In addition, family caregivers’ self-management skills are closely related to health literacy and empowerment to provide care and maintain their own health. Individuals with low health literacy have difficulty dealing with the healthcare system and understanding health-related information. Studies from several countries have shown that low health literacy has a negative impact on health, health- and disease-related behaviors, and the use of health resources [14]. In addition, care recipients and their families need to be encouraged and empowered to take an active role. For this purpose, health information must be imparted with people living with chronic illness and their caregivers as well as empowerment promoted [15].

In the present study, family caregivers’ existing self-management competencies were investigated to gain insight into their health literacy and their level of empowerment with regard to caring for family members as well as maintaining their own health. Turkish origin was chosen as an exemplary migration background for this study because Turkish migrants face particular challenges in connection with care: among other things, this group rarely makes use of institutional care services and relies heavily on family care. It is therefore crucial to understand the self-management skills of caregivers of Turkish origin in order to develop targeted support interventions. The vulnerability that can be associated with a migration background is transferable to other diversity characteristics. It should be noted that the reference to family carers of Turkish origin should therefore only be understood as an illustrative example.

## 2. Materials and Methods

For this qualitative study, semi-structured problem-centered interviews [16] were chosen to adequately capture detailed information on our research topic. The aim of the problem-centered interviews was to collect subjective perceptions, opinions and experiences of the family caregivers about their care situation. For this purpose, a qualitative interview guide was designed, which motivated the interviewees to give a comprehensive account of their own experiences vis-à-vis the research topic [16]. The underlying interview guideline focused on information regarding the relative’s condition, the current care situation, needs and utilization, health literacy, information requirements, personal skills, resources, and empowerment, as well as stress and relief options.

A total of 10 caregivers of Turkish origin, providing care for their family members living with dementia in the Gelsenkirchen/Bottrop area of Germany, were interviewed. We aimed to recruit a group of individuals that was as heterogeneous as possible with regard to relevant characteristics (main caregiver, age of the main caregiver, care recipient, availability of other caregivers, shared living and level of external support). Recruitment took place until theoretical saturation [17] could be reached, so that new findings (including new data) were not expected. A sample description of the participating families is shown in Table 1.

The interviews lasted between 13 and 57 min, with an average duration of 35 min. Variations in interview length resulted from different communication styles and the quantity of information provided by the caregivers. Additionally, the necessity to conduct two interviews via telephone due to the impact of the COVID-19 pandemic contributed to their shorter duration. Despite the shorter duration, the telephone interviews did provide relevant information and did not expand the category system. Recruitment of additional participants was also hindered by the COVID-19 pandemic, but our data analysis confirmed that information saturation had been reached after 10 interviews.

**Figure 1:**
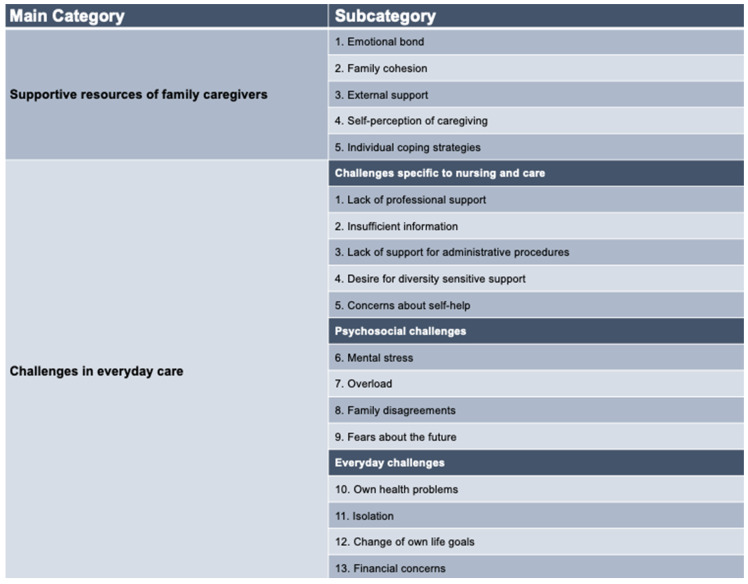
Overview of categories and subcategories.

Interviews were audio-recorded and subsequently transcribed and anonymized. The transcribed material was analyzed using a structuring content analysis following Mayring [18]. The interviews yielded a number of recurring themes, indicating that the information saturation point had been reached and that no further interviews were necessary [19]. The data collection, transcription, translation and analysis were conducted by the project team (Y.Y.-A., T.A. and K.A.) with the help of MAXQDA. The transcripts were analyzed using Mayring’s inductive qualitative content analysis [18]. Following Mayring, categories were first formed deductively. Independently, the project team then assigned individual interview segments to these preliminary categories. Through an inductive expansion of the categorical system, further relevant categories were identified and added in the process (see Figure 1). The categories thus generated, as well as any discrepancies in coding, were discussed in the project team (Y.Y.-A., T.A. and K.A.) until an agreement could be reached [18]. The final version of the code tree included 2 main categories and 18 subcategories (see Figure 1).

## 3. Results

Overall, it was possible to identify five subcategories for the main category *supportive resources of family caregivers* and thirteen subcategories for the main category *challenges in everyday care* (see Figure 1).

### 3.1. Supportive Resources of Family Caregivers

A variety of potentials for a self-determined care situation could be identified through existing supportive resources of family caregivers. These supporting resources can be assigned to the categories “emotional bond”, “family cohesion”, “external support”, “self-perception of caregiving” and “individual coping strategies”.

#### 3.1.1. Emotional Bond

Despite the many challenges and burdens that caregivers face in caring for their family member living with dementia, there are also supportive resources in caregiving. One factor that makes the caregiving situation easier is the emotional bond between caregiver and care recipient. Although living with family members in need of care is described as very difficult overall, efforts are made to respond to situations with ease and love. Thus, families make an effort to treat those in need of care with understanding, care and affection and to accept every behavior whenever possible. They focus on being patient and understanding in dealing with the illness and the family member and on feeling joy and gratitude despite the burden experienced. Consider the following quote from family N (caring daughter, 50 years) to illustrate this point:

*“And yeah, just giving love, passing on love, and getting love from her, because that’s really something that I never, maybe in my childhood yeah, in my youth I didn’t miss from her like that. And now I’m getting that. And that’s an influx of energy that I get for me that keeps me going all week. Even though it’s exhausting to be with her. Yes, I would recommend that to any relative to experience that with them and not always as work. For me it’s not work, it’s taking care of my mother who has always been there for us, always.”*

*(Family N)*

#### 3.1.2. Family Cohesion

Since the care of individuals mainly takes place within the family, sharing care is essential for a healthy care situation. In the interviewed families, the equal distribution of caregiving tasks by family members is perceived as very relieving. This is especially the case among siblings where it is possible to divide care in such a way that each person has an independent area of responsibility. For example, while one sibling is responsible for accompanying the patient to a doctor’s appointment, the other sibling takes care of the household. In addition to this division of care activities, there is also a rotation system such that a different person is responsible for care on the weekend and during the week:

*“So it’s very well organised, I’m also very, very happy that the other siblings also take care of her. My younger sister always does her personal hygiene in the morning. She’s there for about an hour and if something needs to be done around the house, she does it quickly. And our dad often cooks too, so we don’t have to do it. And then my other sister comes in the afternoons on weekdays. And we also have a sister-in-law, thank God, she helps us too.”*

*(Family N)*

#### 3.1.3. External Support

In addition to care within the family, some families also receive external support that is provided outside the family. For example, the interviewed relatives named friends and acquaintances who look after the person in need of care when they themselves are not available or need a break. Neighbors of these families also support the person in need of care as soon as the need arises. This support (or offers of support) through non-relatives relieves the burden of the family caregivers. One respondent noted:

*“My friends—I mean, thanks a lot, if I can’t go on, then they come, then they help me—or if I have to leave, then they come.”*

*(Family Z)*

#### 3.1.4. Self-Perception of Caregiving

In the caregiving situation, family caregivers see themselves and the care recipients in a kind of role reversal. Caregiving relatives are very exhausted by caregiving but would face a conflict of conscience if they did not take on the caregiving. Despite this stress, it is therefore natural for family caregivers to take on caregiving and integrate it into their own daily lives. The following quotation from our data demonstrates this well:

*“But I feel obligated, the way she raised us, that even then I’ll be with Mom as long as possible. That’s actually a lot of fun for me, to still be with her, to be there for her. Yes, she was a very strong personality and now she is our child. And when I think of her as my child, I don’t find it that difficult. Yes, and for me it has been difficult, such a strong personality, because yes, she always guided us, now we have to do it.”*

*(Family N)*

#### 3.1.5. Individual Coping Strategies

Family caregivers have developed various coping strategies. Above all, relatives find physical activities relieving. These include walks, activities in the garden or actual exercise. Occasionally, they also pursue their hobbies, meet with friends or listen to music. In some cases, the exchange with other affected persons is also found to be very helpful:

*“And in between, on the rest days, I do sport, yoga, yoga consciously, to keep my inner peace in harmony, not to freak out. Otherwise, I always take more time out, consciously.”*

*(Family G)*

### 3.2. Challenges in Everyday Care

In addition to the existing resources and associated self-management skills described above, the interviewees also mentioned challenges that family caregivers perceive when caring for a family member living with dementia. These challenges can limit family caregivers’ ability to provide self-directed care and maintain their own health. Figure 1 provides an overview of these difficult-to-manage home care challenges and stresses for the families interviewed.

These aspects can be assigned to the three categories “challenges specific to nursing and care”, “psychosocial challenges” and “everyday challenges”, some of which are mutually dependent and can reinforce or mitigate each other. These caregiving deficits and related needs point to existing barriers for strengthening family caregivers’ health literacy and self-management skills. Self-management skills in family caregivers are influenced by a complex interplay of both available resources that support these skills and challenging caregiving situations. This dynamic combination of resources and challenges results in varying levels of self-management ability among family caregivers.

#### 3.2.1. Challenges Specific to Nursing and Care

##### Lack of Professional Support

Despite the great potential for stress, only a very small proportion of family caregivers make use of professional help. The reasons for this given by the respondents are a lack of information, dissatisfaction with the services on offer and the mostly traditional self-image of providing care within the family. Consider the following quote from our data:

*“Well, we used to do all his cooking, cleaning, you know. We live upstairs and downstairs, we know his habits, we know everything about him, no one else could take care of him. Anyway, we are Turks, as you know, elders are looked after.”*

*(Family A)*

##### Insufficient Information

In some cases, family caregivers have little knowledge about the disease and about existing support and relief services. Family caregivers receive information primarily from their relatives and acquaintances. However, families have doubts about the accuracy and completeness of the information they receive in this way. They would prefer well-founded and more in-depth information, especially about their rights, entitlements and options in care provision. In obtaining and acquiring dementia-specific knowledge on their own, they encounter barriers such as a lack of knowledge about points of contact and language barriers:

*“That’s how it is, I mean, they tell us, the nurse, because she works in a nursing home, they tell us things, sir, they tell us what happened with my wife’s illness, well, they tell us about those who were much worse off.” *

*(Family I)*

##### Lack of Support for Administrative Procedures

Lack of support with administrative procedures is a central theme in the conversations with the families. Families would like to have contact persons who can help them with bureaucratic matters, such as making applications. The families concerned express a need for written information, especially about their rights and entitlements, and for information and services in their native language. One respondent, a 50-year-old caring daughter, stated:

*“But the fact that someone comes, a specialist from the health insurance company, and says: ‘Here, this and this would be your rights’, I’m not informed about that.”*

*(Family G)*

##### Desire for Diversity Sensitive Support

In addition to administrative support, families also lack support in the household and in caring for those in need of care. Institutional assistance, however, would only be perceived as relieving if the services met their specific needs. In particular, the lack of culture- and language-specific care provision is an obstacle to using these services. Since the German language, which is usually learned in later stages of life, fades in the course of the cognitively impairing dementia disease, Turkish-language care and support for those affected is perceived as helpful. Expressing their needs, engaging in confidential health-promoting conversations and enabling better coordination with professionals would make it easier for caregiving family members of patients living with dementia. In addition to language, cultural specifics should also be taken into account; an example is that care which requires physical proximity is provided by caregivers of the same sex, if possible.

*“Whether a stranger could do it now, I don’t know, it has to be a confidant, on a basis of trust, who can also speak Turkish, for the care, I find, I would find that relieving for me.”*

*(Family G)*

##### Concerns about Self-Help

The concept of self-help and offers for self-help are mostly unknown to the affected families. The family caregivers are skeptical about using self-help services because they are in an inner dilemma. On the one hand, they want to talk about everyday caregiving with like-minded people in a self-help group. On the other hand, they fear an additional burden as they would have to deal with the topic of dementia and possible burdens and problems of other affected persons in addition to their already challenging care activities. One respondent stated:

*“I have never experienced such a thing as self-help, I can say that I have never heard of such a thing. We have never been informed about such a thing until now.”*

*(Family I)*

#### 3.2.2. Psychosocial Challenges

##### Mental Stress

The change in a person’s character that occurs as a result of dementia is perceived as very stressful by family caregivers. The loss of abilities and certain valued character traits as well as the increasing forgetfulness and confusion of those affected can be difficult for family members to accept. Particularly challenging for relatives is the dementia-specific aggression of those affected. Misunderstandings between those affected and family caregivers associated with these changes lead to feelings of helplessness, anger and grief:

*“I don’t want to break my mother’s heart, she is 76 years old, I don’t want to break her heart, I don’t want her to be sad, then she cries like a child, and that makes me feel bad. Sometimes it is difficult.”*

*(Family G)*

##### Heavy Workload

Since patients living with dementia can no longer perform everyday tasks independently, they are heavily dependent on outside help. In the families interviewed, tasks such as carrying out hygiene measures, cleaning the home, preparing meals, brushing teeth, and dressing and undressing the affected person are taken over by the family caregivers. In addition, family caregivers provide support for activities outside the home, such as organizing and accompanying visits to the doctor, shopping and outings. Especially when there is no distribution of caregiving tasks by family members, caregivers feel exhausted and overwhelmed. In addition, caregivers often work in addition to their caregiving activities or have care responsibilities in their own family. As a result of the fact that little or no institutional help is sought and other family members also provide little support to the caregiver, the main burden often lies with one person. This caregiver may be exposed to stressors as a result of the heavy workload during caregiving, which can lead to new health problems:

*“It’s overwhelming because we’re all still at home—I still have my parents-in-law there, for example. My father-in-law has bowel cancer, right? And my mother-in-law is 82, too—so she’s only mentally fit, but not physically. Yes, then my husband is still there, as is my daughter, a late-pubescent daughter, who of course also wants me in between. Thank God she helps me a lot, but she also needs my support with certain things.”*

*(Family N)*

##### Family Disagreements

In addition to the already stressful care situation for family caregivers, family disagreements related to caregiving present an additional challenge. Different views and opinions on the illness of the affected family member and the associated care lead to conflicts between family members. An unequal division of care-related labor is sometimes perceived as the most significant problem. As some family members do not want to acknowledge the dementia, may assess the care needs of the affected person differently or may live further away, care is usually the responsibility of only one person. The support by other family members is thus often perceived as insufficient. However, when other family members assist with care, disputes about this care may arise nonetheless because ideas about the kind of care needed may vary, the responsibilities of caregivers are unclear, and tasks are delegated within the family without sufficient communication.

*“There have already been big wars with us—especially the girls, they don’t go along with it. They’re totally against it. One of the older ones says it can’t be, my mum won’t get sick for another ten years. It’s not an illness.”*

*(Family H)*

##### Fears about the Future

Psychosocial stress in caregiving is also rooted in strong fears about the future among family caregivers. Family caregivers fear that the health of the affected person will deteriorate in the future. This gives rise to fears of an increased need for care and increasing excessive demands. This is followed by thoughts about what the care situation will look like in the future and raises questions about taking over and sharing care within the family when the need for care increases. The uncertain development of the illness and the accompanying worries about a future care situation can lead to feelings of powerlessness, including trouble sleeping, for some respondents:

*“It hurts every day to know this, that it’s getting worse and worse.”*

*(Family K)*

#### 3.2.3. Everyday Challenges

##### Own Health Problems

In addition to psychosocial and emotional concerns, family caregivers also suffer from physical stress. First and foremost, respondents complain of headaches, feeling fatigued and lack of sleep. Added to this are their own illnesses such as heart disease, diabetes and high blood pressure. These ailments are perceived by those affected as a heavy burden and restriction in their personal everyday life as well as in their daily care routine and can lead to exhaustion or even forcing them to quit their own jobs. Family Z explained (50-year-old caring daughter):

*“Not so good. Yes, what do you mean, not so good? I think I had stomach surgery in 2007. I’ve had problems ever since. I have a constant vitamin deficiency, but it’s not just this operation, it’s everything I’ve been through over the years, I’ve lived and I work really hard. And I’m also getting older and older. At some point the body says, stop, no, now you have to take it easy.”*

*(Family Z)*

##### Isolation

The time-intensive care as well as the obligations in one’s own everyday life are often accompanied by an abandonment of leisure activities and a reduction in personal contacts. The relatives lack the time and energy to maintain social relationships in the non-family environment and to make use of offers for social participation. The isolation that results from this leads to loneliness and dissatisfaction and represents a further burden for the family caregiver:

*“You know, I am very lonely here.”*

*(Family I)*

##### Change in Own Life Goals

Time-intensive caregiving has a significant impact on the caregiver’s own life. Among other things, changes in their lives as a result of caregiving are expressed in the abandonment of their own occupation. This happens especially in the case of an increased need for care at a later stage of the illness. Caregiving relatives also find little time for hobbies and other activities that they enjoyed in the past and that can act as coping strategies during the stressful caregiving process. Individual needs, visions and life plans also suffer as a result of the caregiving activity. Thus, personal plans, such as the desire to return to the home country or to marry, are postponed or even abandoned.

*“I don’t know, my conscience didn’t allow it, maybe I could have done it like the others. I could have moved into my own apartment, but I didn’t. Maybe because I’m so conscientious, I think. It was hard, I took something from my own life and gave it to my mother. I don’t regret it, but I am very exhausted.”*

*(Family Z, translated)*

##### Financial Concerns

Care within the family can lead to financial difficulties or exacerbate already existing problems. In order to meet the care needs and focus holistically on caring for the sick family member, family caregivers often give up their jobs or reduce their working hours. This care-related career change puts a strain on the family’s finances and presents the caregivers with major challenges in decision-making when it comes to reconciling the time spent caregiving by working. The financial burden can limit family caregivers’ opportunities for relief and recreation, as they can no longer take advantage of leisure activities to the extent they are accustomed to; often, a vacation is not possible.

*‘I need money, do you know how much money the two of us get, we get 1300 liras, we have 450 liras 55 liras rent, we pay a hundred liras for electricity, there is telephone money. The money for food and drink, everything is expensive. We can hardly afford it, we haven’t been on leave for two years.’*

*(Interview E1, Family N.S.)*

## 4. Discussion

The aim of the present study was to gain insight into the existing self-management competencies of family caregivers of Turkish origin for relatives living with dementia in home care. In examining these competencies and the associated health literacy and empowerment of family caregivers, existing supportive resources available to family caregivers for a self-determined caregiving situation became apparent in the interviews. A strong emotional relationship, family cohesion, support outside the family, the self-image of caregiving and personal coping strategies can ease a stressful care situation for family caregivers. From these supportive factors, family caregivers draw energy for the long-term care of their family members. In addition, they can use these resources to develop and implement approaches for self-determined care management as well as for their own relief. The existing support factors can promote the development of their self-management skills and strengthen family caregivers in organizing care independently while protecting their health.

In addition to these existing self-management skills, however, this study also identified unresolved challenges that make it difficult for family caregivers to manage the caregiving situation independently.

Care and caregiving-specific challenges such as insufficient information or the desire for diversity-sensitive care, psychosocial challenges such as being overwhelmed and fearing the future, and everyday challenges such as changing life goals and financial concerns contrast with resources that can promote the development of self-management skills. Care and support-specific challenges result primarily from low use of care services in families. The non-utilization of external support is particularly due to a lack of information about dementia and existing offers. In addition, the existing services are insufficiently adapted to the needs of the target group, so they are not perceived as relieving by the families. The findings support those of previous studies, which also show that the often exclusively intrafamilial care is due to existing knowledge and care deficits regarding institutional support options [20,21]. Psychosocial challenges in caregiving result mainly from the high care effort in combination with the emotional stress due to the family closeness of the ill person to the caregiver. In particular, the life challenges of family caregiving are perceived as a major burden by family caregivers. Due to changing lifestyles, subsequent generations of migrant workers in particular are adapting to the less collectively oriented lifestyles of the majority population [22]. In the future, educational advancement may also no longer be compatible with the unconditional and self-evident care provided by family caregivers [23]. The dilemma between one’s own self-development on the one hand and family-centeredness in caregiving on the other hand can be observed especially in the third generation [24] and will possibly represent another lifeworld challenge for family caregivers.

Due to the diverse potentials and challenges, the interviewed family caregivers of Turkish origin show a heterogeneous state of self-management in caregiving and maintaining their own health. Some family caregivers are able to provide informed and self-determined care for their family member. These caregivers are particularly characterized by the presence of supportive resources. For example, they have a close and solid relationship with their family member in need of care and strong family cohesion and receive extra-familial support in addition to intra-familial support. In addition, self-awareness of caregiving and the development and implementation of individual coping strategies can be factors that activate the self-management skills of family caregivers. By using existing resources, a considerable number of caregivers appear to be able to cope with the care-specific, psychosocial and everyday challenges associated with caregiving and thus realize a caregiving situation that is as autonomous and health-preserving as possible. However, it remains questionable how long family caregivers can sustain this without risking their own long-term health, especially when they frequently refrain from seeking external support.

Family caregivers and families who have few or no supportive resources could be studied. These family caregivers generally have low self-management skills. Information deficits, family disagreements, lack of support within and outside the family, and lack of individual coping strategies characterize the caregiving situation in this group. Deficits in strengthening resources can lead to inadequate responses to caregiving challenges. Thus, the majority of caregivers are unable to “emerge from a state of passive suffering” [11] and lack the ability and (self-)confidence to actively and self-determine their own health or illness situation, which may change as a result of taking on caregiving [11]. Rather, the results of the interviews show a high need among these family caregivers for support in caring for relatives, up to and including support in relieving themselves of unresolved challenges such as excessive demands and psychological stress.

For the families concerned, it is highly relevant to recognize and promote existing resources in order to be able to cope with the challenges of caregiving. The results of this study make clear that interventions need to be developed that strengthen supportive resources and make targeted use of the resources of the target group. In addition to concepts to build and promote self-management, caregiving and information-sharing services are essential. Self-management skills should be taught in a manner tailored to the specific needs of family caregivers, enabling them to effectively apply these skills to address challenges. Care must be taken to ensure that services are tailored to the different needs and lifeworlds of the families concerned in order to be accepted by them. These requirements for the development of support structures apply to family caregivers, regardless of whether they have a history of migration or not. Tezcan-Güntekin and Razum also state that people affected by dementia have heterogeneous needs depending on their biographies, which can only be addressed through a person-centered and diversity-sensitive approach [25]. The development and implementation of individual approaches with regard to different living environments; family and care constellations; and, in particular, existing resources should be taken into account in future projects and planning to promote self-management. Interventions to support family caregivers should follow an individual and participatory approach that identifies and specifically promotes existing resources. Concepts for building and promoting self-management skills should always focus on the autonomy and active role of relatives [11]. This is the only way to maintain home care for people in need of long-term care, which consists of a combination of self-determined, family and professional support.

The present study has several strengths that contribute to its relevance and originality. First, family caregivers in families of Turkish origin were recruited and interviewed for this study. This target group is a difficult population to reach for research, so the inclusion of this group is of great importance [26]. The inclusion of families of Turkish origin adds to existing knowledge about family caregivers, as there has been limited evidence on this specific population. Another strength of this work is the comprehensive presentation of support options and challenges from the perspective of affected families. By using appropriate methods and instruments, it was possible to examine a wide range of factors that have both positive and negative effects on the caregiving experience. This comprehensive approach provides a detailed understanding of the specific needs and challenges of family caregivers in families of Turkish origin. However, there are also some limitations to consider. It should be noted that the recruitment and interviewing of family caregivers in families of Turkish origin allows only limited comparisons between people with and without a history of migration in terms of supportive resources and challenges. It can be assumed that the challenges in everyday care described by families of Turkish origin are also present in families with a different background or without a history of migration. Furthermore, this study focused exclusively on the care of people living with dementia. Therefore, it remains unclear to what extent the results are transferable to other diseases. Future studies should include a variety of cultural backgrounds and caregiving situations to gain a more comprehensive understanding of the diverse experiences and needs of family caregivers. Another aspect that can be considered a limitation is the narrow geographic scope of this study. The interviews were conducted in only one German state, i.e., North Rhine-Westphalia. However, it is unlikely that the experiences and challenges of family caregivers in families with Turkish origin in other regions or federal states are substantially different.

## 5. Conclusions

People of Turkish origin continue to face numerous challenges and barriers in caring for family members living with dementia. However, they have supportive resources that play a vital role in overcoming these challenges. It is crucial for the supportive resources of family caregivers to be strengthened, enabling them to provide care in a self-directed and health-promoting manner. Long-term support structures need to be created that aim to promote the use of resources and self-management skills of family caregivers. Moreover, it is crucial to consider both the individual resources and the social and cultural diversity of the families involved. Taking these factors into account is necessary for effective approaches and interventions that can offer meaningful assistance. The challenges faced by family caregivers with a history of migration are similar to those affecting caregivers without a history of migration. As such, there is a pressing need to offer demand-oriented services and support with a particular emphasis on diversity sensitivity. By doing so, the unique needs of these caregivers and their families could be better addressed.

## Figures and Tables

**Table 1:** Sample description of the ten caregivers of family members living with dementia.

Family ID	Main Caregiver	Age of Main Caregiver	Care Recipient	Availability of Other Caregivers	Shared Living	External Support
Family S	Mother	77	Son	No	Yes	No
Family H	Daughter	50	Mother	No	No	No
Family K	Husband	53	Wife	Yes, several family members	Yes	No
Family A	Wife	66	Husband	Yes, daughter	Yes	No
Family E	Wife	78	Husband	Yes, daughter	No	No
Family Ö	Husband	58	Wife	No	Yes	No
Family C	Daughter-in-law	43	Father-in-law	Yes, multiple family members	Yes	No
Family G	Daughter	50	Mother	Yes, father (husband)	No	Support group, twice daily care provider
Family N	Daughter	50	Mother	Yes, several family members	Yes	No
Family Z	Daughter	49	Mother	No	Yes	Twice daily care service

## Data Availability

Given the potentially disclosive nature, entire interview transcripts will not be made publicly available. They will be deposited at Bielefeld University, and reasonable requests for secure research access will be considered. Please contact yuece.yilmaz-aslan@uni-wh.de.
